# How Does Part-Time Farming Affect Farmers’ Adoption of Conservation Agriculture in Jianghan Plain, China?

**DOI:** 10.3390/ijerph17165983

**Published:** 2020-08-18

**Authors:** Xin Yang, Yiming Sang

**Affiliations:** College of Land Management, Huazhong Agricultural University, Wuhan 430072, China; sangyiming@webmail.hzau.edu.cn

**Keywords:** adoption behavior, conservation agriculture (CA) technology, farmers, logistic model, part-time farming

## Abstract

Part-time farming has been suggested by scholars to play an important part in farmers’ decision making, but seldom empirical evidence has been done on the field of conservation agriculture (CA) technology adoption worldwide. Based on the field survey data of 433 farmers in Jianghan Plain, China, this paper estimate the impact of part-time farming on farmers’ adoption of CA technology by applying the multivariate logistic model. The results show that 91.92% of the farmers adopted CA technology. Part-time farming had a highly significant positive influence on the likelihood of adoption. Moreover, the impact degree increased along with the deepening of part-time farming. In addition, farmers’ adoption behaviors were affected by gender, contracted land area, economic welfare cognition and social welfare cognition. Our results help to understand farmers’ complex decision-making on farmland and to promote the sustainable development of agriculture in Jianghan Plain. A somewhat targeted approach to design policies to support technological, policy and institutional interventions to encourage farmers to engage in part-time farming are recommended, especially in areas that share similar edaphic and climatic characteristics with Jianghan Plain.

## 1. Introduction

Since the “reform and opening up” policy, China has made great achievements in agricultural development. However, due to the long time high intensity development mode of farmland, its soil fertility has been seriously deteriorated during the past few decades, which lead to the quality of agricultural products decline, biodiversity loss and environment deterioration [1]. So, it is of vital significance to promote sustainable development of agriculture in China [2]. As an important technology for farmland quality protection, conservation agriculture (CA) technology includes straw returning to field, shrimp-fish culture, no-till and less-tillage on farmland, pesticides control technique, deep tillager, etc. [3]. This technology not only has the functions of improving farmland productivity, but also offers environmental benefits of reducing energy consumption and curbing farmland degradation [4,5]. Therefore, the National Agricultural Modernization Plan (2016–2020) issued by the central government of China in 2016, who decided to accelerate the promotion and application of mechanized technologies such as deep-sowing land preparation, and straw returning to the field by mechanization. The Agricultural Mechanization to Promote Agricultural Green Development Work Plan proposed that conservation agriculture (CA) technology should be provided with subsidies to increase their financial support.

Currently, the phenomenon of part-time operation is common in agricultural production in China. It is of great significance to study the impact of part-time farming on the adoption of CA technology. Research in this field is mainly focusing on two aspects. (1) Research on the influencing factors of CA technology adoption behavior. The research results of Fei et al. showed that factors such as the proportion of non-agricultural income, expected income, years of land transfer and the role of neighborhood demonstrations significantly affect farmers’ willingness to adopt CA technology [6]. Cai et al. used the Probit model to find the main factors affecting the adoption of CA technology are the awareness of the CA technology’s role, the status of the farmers’ part-time farming, the years of the farmers’ education and whether the farmers are village cadres [7]. Li et al. found that farmers with a lower proportion of agricultural income and higher family income adopt CA technology at a higher level [8]. Prokopy et al. mentioned that from the perspective of technology demand, farmer characteristics, management characteristics, farmland characteristics and external environment are the main factors affecting the adoption of CA technology [9]. Francis et al. mentioned in the study of the influencing factors of CA adoption in Australia that CA adoption is a process of information intensive and learning intensive, so we can broaden their access to information by improving the level of part-time farming [10]. In addition, Duncan et al. provide a theoretical basis for the positive impact of part-time farming on the adoption of CA technology by mentioned that non-agricultural activity is one of the influencing factors for CA adoption [11]. (2) The impact of a single factor on farmers’ adoption of CA technology. Research by Gao et al. showed that farmers’ willingness to adopt CA is affected by the frequency of extreme weather events [12]. Ma et al. found that the effect of government subsidies significantly promoted the adoption of CA technology among fruit farmers [13]. Duncan and Ben suggested education, be it specific or general, commonly correlates positively with the adoption of conservation agriculture practices [11]. There are many factors that affect farmers’ adoption of CA technology, among which part-time farming is one of the important factors that cannot be ignored. With the economic and social development, the degree of part-time farming has been deepening, and the number of farmers who have a part-time job has increased rapidly [14]. The response of farmers with different part-time jobs to the external environment such as the market, policies and the farmland using methods caused by them will also be different [15]. In this context, Yang and Fan showed that part-time farming had a significant direct or indirect negative impact on the rural ecological environment [16]. Yang et al. found that the proportion of farmers’ investment in farmland quality protection decreased with the deepening of part-time employment [17]. Research by Zhu and Li found that with the adjustment of the industrial structure, the degree of part-time employment has an obvious influence on the adoption of new technology [18].

To sum up, the above research has provided a solid foundation for this paper, however, the following shortcomings still exist: (1) In the existing research, with the deepening of the degree of part-time farming, how to impact the farmers’ adoption of CA technology has not reached a consistent conclusion. (2) Most of the existing studies have taken the Yellow River Basin and Northeast China as the study areas, using dry farming as the main farming method. There are few related studies in other areas, and the farming patterns in different areas may be different. (3) Part-time farming is one of the influencing factors for the adoption of CA technology. Although existing literature considers the influencing factor of part-time farming when studying CA, which is only as one of the variables. The mechanism of its impact on CA was not studied separately. In this paper, we used the control variable method to deal with other variables, in order to study the part-time farming emphatically. Therefore, based on the data collected from field investigation, this paper studied the impact of different part-time farming situation on their CA technology adoption, so as to provide a theoretical basis for the government to design a more scientific CA technology promotion policy.

## 2. Research Hypothesis and Research Method

### 2.1. Research Hypothesis

The impact of the part-time farming for farmers on the adoption of CA technology proposed in this paper includes the following two research hypotheses:

**Hypothesis 1 (H1).**  *Part-time farming has a significant positive impact on farmers’ adoption of CA technology.*

Part-time farming has changed the endowment of farm households and the input structure of agricultural production [16]. Farmers’ participating in non-agricultural production will not only change their constraint level of agricultural production resource endowment, but also affect their agricultural production goals [19], Moreover, working members from part-time farming family lives in cities all the year round have more opportunities to understand the role of CA technology. They look at CA technology from a development perspective and are more inclined to adopt CA technology that are both more beneficial to environmental protection and family income [20].

**Hypothesis 2 (H2).**  *The possibility of CA technology adoption increases when the part-time farming goes deeper.*

Part-time behavior is an optimal allocation of labor resources in one family. Employment of labor in agricultural and non-agricultural fields both can promote the maximization of family income [21]. The increase of farmers’ income provides an economic basis for adopting CA technology. Compared with pure farmers, part-time farmers have more money to invest in CA technology. Therefore, It was hypothesized that those with deeper part-time farming are likely to have greater potential to benefit from the CA technology and are therefore more likely to adopt it.

### 2.2. Research Method

#### 2.2.1. Variable Definition

CA technologies include no-till and reduced tillage, straw and topsoil treatment, no-till seeding and weed and pest control, whose adoption behavior is affected by multiple factors [22]. Based on the number of types of CA technology adopted, we divided the dependent variable into 6 categories and assigned values separately. As far as the degree of part-time work of farmers is concerned, it generally refers to the degree to which farmers are engaged in both agricultural and non-agricultural production and get paid both from sections [23]. According to the definition of the degree of part-time farming in previous literature [24,25], part-time farming degree is mostly expressed by the proportion of non-agricultural production income to the family’s total income. Therefore, farmers were classified into four different types in this study: full-time farmers (non-agricultural accounting for 0–10%), I part-time farmers (non-agricultural accounting for 10–50%), II part-time farmers (non-agricultural accounting for 50–90%) and non-agricultural farmers (non-agricultural accounting for 90–100%). According to the existing literature in this research field [17,26,27], this paper divided the other factors that affect the adoption of CA technology into three aspects: the farmer’s individual characteristics, the farmer’s family characteristics and the farmers’ cognition of CA technology. The individual characteristics of farmers include gender, age, education level and whether they served as the leaders in the village or not (village cadre). The family characteristics include the number of permanent agricultural workers, the health status of family members and the area of contracted land. The cognition includes economic welfare cognition, social welfare cognition and ecological welfare cognition. Last but not least, we also tried to figure out the heterogeneity by defining the variable of region.

The specific definition and description of each variable are shown in Table 1.

#### 2.2.2. Multivariate Logistic Model

The CA technology varies with the different edaphic and climatic characteristics of areas where the study was carried out. Specifically, there are 5 different kinds of CA technologies (straw returning to field, shrimp-fish culture, no-till and less-tillage on farmland, pesticides control technique and deep tillager) that were widely implemented in Jianghan Plain. 

In this study, there are two kinds of farmers were first recognized as “adopting CA technology” and “not adopting CA technology”. Furthermore, we classified the “adopting” into 5 subgroups according to the amounts of CA technology adopted by the respondents. Therefore, this study defined *y* as “how many kinds of CA technology have your family adopted?” to express the farmers’ degree of adoption. A multivariate logistic model is constructed to study the impact of part-time farming on their adoption degree of CA technology. The model is expressed as follows:

(1) $$P=F(y)=\frac{1}{1+e^{-y}}$$

In formula (1), P represents the probability of farmers adopting CA technology with different degrees of part-time farming. y is the adoption degree, whose changes can be explained by the combination of explanatory variables ($x_{1}$ …$x_{14}$). Hence, the formula can be described as:

(2) $$y=\beta_{0}+\beta_{1}x_{1}+\beta_{2}x_{2}+\beta_{3}x_{3}+\ldots +\beta_{14}x_{14}+\varepsilon$$

In formula (2), *β_i_* is the coefficient corresponding to each explanatory variable, $\beta_{0}$ is the constant of the regression equation and  ε is the random error. $x_{1}$ is full time households and $x_{2}$ and $x_{3}$ are I part-time households and II part-time households. $x_{4}$ …$x_{13}$ represent gender, age, education level, village cadre, number of agri-labor force, health status, contracted area, economic welfare cognition, social welfare cognition and ecological welfare cognition respectively. Despite sharing the similar edaphic and climatic characteristics, region is introduced into the model to address the impact caused by the location of farmers, named as $x_{14}$. After proper treatment of formulas (1) and (2), the binary logistic model is described as follows:

(3) $$n\frac{P}{1-P}=\beta_{0}+\beta_{1}x_{1}+\beta_{2}x_{2}+\beta_{3}x_{3}+\ldots +\beta_{14}x_{14}+\varepsilon$$

#### 2.2.3. Flow Chart 

**Figure 1:**
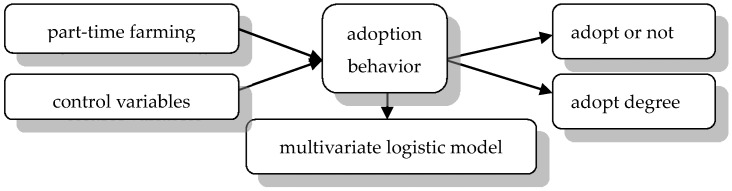
The flow chart of this paper.

The framework (Figure 1) of this study can be stated as: the farmers’ adoption of CA technology is not only influenced by part-time farming, but also by other variables, such as gender, age, cognition degree, etc. Therefore, firstly, Model 1 is introduced to examine the impact of part-time farming on farmers’ adoption. Then, Model 2 is applied to test the validity of the effect from part-time farming by controlling the other dependent variables. Particularly, by applying the multivariate logistic model, we could use the “adoption degree “ rather than “adopt or not” to describe farmers’ adoption behavior, which will explain the impact imposed by part-time farming better.

## 3. Research Area and Data Sources

### 3.1. Research Area

Located between 29°26′–31°37′ N and 111°14′–114°36′ E, the Jianghan Plain is an essential part of the middle and lower reaches of the Yangtze River Plain and an important food grain region of China. With an area of more than 48 × 10^5^ hm^2^ (note: 1 hm^2^ = 0.01 km^2^), the Jianghan Plain has abundant surface water systems including interweaving rivers, lakes, channels and ditched ponds. It has flat and fertile soil, which is mainly made up of fine sand, silt and clay. The study area has a subtropical monsoonal climate, the average annual precipitation in Jianghan Plain is about 1100–1300 mm and annual temperature ranges between 15 and 17 °C. Due to the massively rapid urbanization process and intensive pattern of farming, the quality of soil fertility in this area has severely declined during the past decades. Therefore, CA technology, which offers tremendous environmental benefits by reducing energy consumption and curbing farmland degradation, has attracted the attention from governments in the Jianghan Plain. Five different kinds of CA technologies (straw returning to field, shrimp-fish culture, the different edaphic and climatic characteristics of no-till and less-tillage on farmland, pesticides control technique and deep tillager) that adapted to the edaphic and climatic characteristics of the Jianghan Plain are widely implemented in this area.

With the rapid development of economy in recent years, there are a tremendous amount of farmers that choose to be employed in the non-agricultural industrial factories. Serving as the important national important bases for grain production, Jingshan City and Yicheng City are experiencing a process of rapid CA technology promoting and up trending part-time farming [28]. Hence, the survey too is placed mainly in Jingshan and Yicheng, two county level cities of Hubei. Jingshan City is located in the transition zone from the hills in central Hubei Province to the Jianghan Plain. With fertile soil and a rich spring water resource, Jingshan is the birthplaces of Chinese farming culture and unique production bases of high-quality rice (named as Guobao Qiaomi). There are about 10 × 10^4^ hm^2^ farmland in Jingshan, and the agricultural products are mainly rice, cotton and oil in the Hubei Province [28]. Jingshan City has been the main agricultural product production area at the national level since December of 2012. Focusing on protecting farmland, stabilizing the production and supply of agricultural products and ensuring national grain and food security are the three goals set by the central government [29]. According to the statistics of 2018, the mechanized pulverization of straw returned to the fields in Jingshan reached 1585 thousand mu (note: 1 mu = 0.067 hm^2^). Additionally, the utilization rate of fertilizer of crushed straw returned to the fields reached 81.60%. In addition, 200 sets of straw returning machines, silage grinders, straw baling machines and other straw utilization tools were added.

Yicheng City is located in the northwestern part of Hubei Province and in the middle reaches of the Hanjiang River, which is a national grain producing base in central China. Its entire terrain pattern is “three mountains, two waters and five fields”. Its agricultural development emphasizes “improving agricultural quality, efficiency and competitiveness” and “improving sustainable development capacity”. It takes “stabilizing the yield, adjusting the structure, changing the mode, and improving the quality and efficiency” as the main development direction [30]. CA technology has developed rapidly in Yicheng during the past two decades. Shrimp-fish culture was highly promoted by governments in Yicheng. Agricultural science and technology demonstration parks used for “rice and shrimp co-cultivation” are vigorously constructed in Yicheng, with one of the largest park taking up approximately 80 hm^2^. Moreover, it proposes to improve the cultivated land quality of 1.33 × 10^4^ hm^2^, and reduce the use of fertilizer by 10% annually during 2017–2019.

### 3.2. Data Source

The main contents of the questionnaire included the family socioeconomic characteristics, the cognition of CA technology and the adoption of CA technology of farmers. The data came from the field sampling survey of farmers in Jingshan City and Yicheng City, Hubei Province in January 2020. According to factors such as regional social and economic development conditions, rural environmental conditions and main characteristics of farmers, Xinshi Town, Sunqiao Town of Jingshan City and Liushui Town, Nanying Township, Longtou Township and Yancheng Street of Yicheng City, totally six towns, were selected as the survey area. Additionally, according to the size of towns, 2–4 villages were randomly selected in each town. A total of 454 questionnaires were distributed, and 433 effective questionnaires were collected, with a valid rate of 95.4%.

## 4. Results and Discussion

### 4.1. Socioeconomic Characteristics

Table 2 describes the basic socioeconomic situation of the interviewed farmers. From the perspective of gender, most of the sample farmers were men, accounting for 71.8% of the sample, and women accounting for 28.2%. In terms of age, only 4.6% of farmers were aged 40 and below, of those aged 41–50 accounted for 20.3% and 75.1% of those were aged 50 and above. It can be seen that the current rural agricultural labor force was mainly middle-aged and elderly. From the perspective of the education level, 51.5% of the farmers had primary school education and below, which was close to half of the total sample. Farmers with junior high school education accounted for 41.8%, and high school and above accounted for only 6.7%, which reflects that farmers’ educational level is generally low. The age structure and education level of farmers directly affect their labor ability and information receiving ability, which in turn has an impact on the adoption of CA technology. Judging from the identity of farmers, 91.2% of farmers are ordinary farmers, and only 8.8% of them have served as village cadres. From the perspective of the annual family income of farmers, the annual disposable income of a rural family in Hubei Province is about 49,173 yuan in 2019. In the sample, 29.8% of the households had a family income of 20,000 yuan or less, 26.8% of the households had a family income of 20,000–40,000 yuan and 19.2% of the households had a family income of 40,000–60,000 Yuan. The average value of the sample data was about 40,000, indicating that the family income level of most of farmers is limited, and their economic conditions play a certain role in restricting the adoption of CA technology. Judging from the scale of contracted land, there were 180 households with more than 12 acres of contracted land, accounting for 41.6% of the total sample, indicating that most farmers had contracted a land scale for CA technology.

### 4.2. Part-Time Farming Analysis

Among the sample farmers, pure farmers accounted for 47.3% of the total sample farmers, 16.9% were agricultural and part-time households, the agricultural and part-time households accounted for 16.9%, the non-agricultural and part-time households accounted for 21.9% and the non-farmers accounted for 13.9% in Table 3. According to the statistical data, with the strengthening of the degree of part-time employment, the annual income of rural households has increased, showing a trend of simultaneous growth with the degree of part-time employment. However, the contracted land scale has an obvious trend of decline with the deepening of the degree of part-time employment. Additionally, the level of education and the proportion of people over the age of 50 had not changed significantly with the situation of part-time work. The socioeconomic situation of different types of part-time farmer family is as follows:

### 4.3. Famers’ Adoption of CA Technology

#### 4.3.1. Cognition Degree of Farmers’ CA Technology

Most of the interviewed farmers have heard about the CA technologies such as straw mulching technology, shrimp and rice co-crop technology and pesticide and fertilizer reduction. More than half of the farmers think that CA can improve soil and reduce greenhouse gas emissions. Of the respondents 31.2% were more concerned about the policies related to CA technology, but 68.8% of the farmers still did not pay much attention to the related policies. In addition, 81.1% of the farmers said that they had not participated in the training of CA technology. Half of the farmers believe that the local government has not promoted CA technology vigorously, indicating that the promotion of CA technology still needs to be strengthened. Some farmers said that although conservative tillage technology is beneficial to improving land quality and social development, it cannot bring good economic income for farmers themselves, which is also become one of the reasons why conservative tillage technology is not adopted.

#### 4.3.2. Farmers’ Adoption of CA Technology

The results showed in Table 4 that 398 respondents adopted CA technology, accounting for 91.92% of the total sample, which is much higher than the number of non-adopted farmers. Among them, the average age of farmers who adopted CA technology was over 50 years old, the annual household income and contracted land scale were slightly higher than those who did not adopt CA technology. The main reasons why farmers were unwilling to adopt CA technology included the reducing agricultural income, the uneasy technology operation, the small farmland area and the lack of labor force.

### 4.4. The Impact of Part-Time Farming on Farmers’ Adoption of CA Technology

In this study, the SAS 17.0 (Statistical Analysis System, Raleigh, NC, USA) was applied to analyze the survey data. The variance inflation factor of each independent variable was lower than 10, so there was no multicollinearity problem among the independent variables in the multivariate logistic model. Moreover, only one independent variable (part-time farming) was taken into Model I to figure out the impact of part-time farming on farmers’ adoption of CA technology. To get a more accurate result, we ran Model II to add all the other dependent variables as the control variables on the basis of Model I. Empirical results obtained from estimating the above two models are summarized in Table 5.

According to Table 5, all the explanatory variables in Model I and Model II have the expected signs and are statistically significant. Both Model I and Model II in Table 5 show that part-time farming had significant impacts on the adoption of CA technology, which is consistent with the findings of Duncan et al. [11], Ma et al. [13] and Fei et al. [6]. In Jianghan Plain, rural households with farmland have the desire to improve their agriculture income and thus are more likely to try CA technologies. Therefore, the results of our analysis accept the hypothesis 1. However, the results of Model I indicate that, although full time farmers, I part-time farmers and II part-time farmers all have higher possibility when comparing with non agricultural farmers, the coefficients of the former 3 groups are in decline while the part-time farming goes deeper. Therefore, we had to reject hypothesis 2.

Pannell et al. proposed that landholders’ adoption of CA is a dynamic learning process that depends on a series of personal social, cultural and economic factors, as well as the characteristics of CA itself [1]. Hence, we introduced Model II to avoid the impact from other dependent variables. Unlike Model I, Model II shows that after we control for the farmer’s gender, age and level of education, whether serve as a village cadre or not, number of agricultural labors, health condition, area of contracted land and market access, farmers’ welfare cognition degree, all the other three groups of farmers have a higher probability of adopting CA technology than non agricultural households. Specifically, the coefficients of full-time households, I part-time households and II part-time households are 0.698, 0.670 and 0.824 with their confidence interval at a *p*-value lower than 0.10, 0.10 and 0.05 respectively. The above results in Model II of Table 5 in indicated that no significant different probabilities of CA adopting were found between the full-time and I part-time farmers. However, II part-time farmers had the highest level of CA technology adoption. According to our comparison between I part-time households and II part-time households, we found that farmers’ adoption rate of CA technology has increased when their part-time farming goes deeper, which is consistent with the research conclusions of Li et al. [8].

There are two reasons account for the above phenomena: (1) On the one hand, the rapid development of the rural non-agricultural industry has attracted more young and middle-aged rural laborers to engage in non-agricultural employment [7]. The increase in household income of farmers makes families are better to bear risk, which give financial support for farmers to adopt CA technology. (2) On the other hand, experiencing the lives in cities, part-time members have more access to information about the welfare value of CA technology [10]. Therefore, for the sake of the next generations and the further development of our nation, they prefer to adopt more advanced CA technologies, such as covering the straw after harvesting, adopting no-till and less-tillage on farmland [31,32]. Additionally, their preference can be transmitted to their farming members of the family to persuade them to adopt the CA technology [33]. Consequently, the analysis of the results of Model II accepted hypothesis 2 on the condition that non-agricultural income accounting for no more than 90% of the whole income.

As for the control variables of farmers, the coefficient of gender passed the significance test at the 1% level, and the direction of the impact was negative. It suggests that men are more receptive to CA technology than women. Households with more contracted area of farmland tend to have higher incentive to adopt CA technology. Within our sample, more contracted area of farmland provides farmers with the foundation to adopt new CA technologies. Moreover, the uptake of CA technologies is often influenced both by the farmer’s cognition of economic and social welfare brought by the CA technologies. According to Pannell et al. [1], farmers that have a higher level of cognition are more attracted by CA’s non market benefits. Within our sample, the cognition degree of economic and social welfare was strongly associated with the adoption behavior of the farmers. As the ecological civilization construction has reached remarkable progress and the environment condition in many rural areas has been improved [34], farmers are satisfied with the status quo, which leads to the insignificant impact of ecological welfare cognition. Moreover, the negative coefficient on regions indicates farmers in Yicheng have a higher probability of adoption CA technology than those in Jingshan. Yicheng is well known for its Yicheng shrimp-rice industry, which has strict limits on the input of presidents and chemical fertilizer during its growing season. 

On the whole, the above findings suggest that part-time farming has significantly positive impact on farmers’ adoption of CA technology. Additionally, their decisions will also be affected by gender, area of contracted farmland, cognition degree of economical and social welfare. These results are keeping with the previous research in this field [1,8].

## 5. Conclusions and Policy Implication

### 5.1. Conclusions

In recent years, the issue of CA technology adoption has attracted the attention from both governments and scholars. Based on the survey data of 433 households in the Jianghan Plain, this paper conducted an empirical analysis that enriched our understanding of the complex effect of part-time farming on the adoption of CA technology by showing how these effects differentiate among specific groups of part-time farming household types. The conclusions are summarized as followed: (1) Most farmers had willingness to adopt CA technology, and 91.92% of them had adopted it. (2) After we controlled the other dependent variables, such as gender, contracted area, whether they serve as a village cadre or not, etc., part-time farming had a significant impacts on the farmers’ probability of CA technology adoption. Specifically, compared with non agricultural farmers, farmers of full-time, I part-time and II part-time all had a higher probability to adopt CA technology, their coefficients were significant at the 10% (full-time households), 10% (I part-time households) and 5% (II part-time households) level respectively. (3) There was no significant difference between the full-time and I part-time farmers on the probability of CA adopting. However, II part-time farmers had the highest level of CA technology adoption, which indicates that part-time farming had effectively strengthened farmers’ adoption of CA technology. (4) Additionally, male farmers were more willing to adopt CA technology, and the contracted land area, economic welfare cognition and social welfare cognition had significant positive impacts on farmers’ adoption behavior of CA technology.

However, limitations still exist: (1) Part-time farming and the adoption of CA in different areas vary, so the representation level of the sample needs to be improved. (2) This paper used the types of adopted technology to characterize the degree of farmers’ adoption on CA technology, which can be improved by adding the adoption area in the future.

### 5.2. Policy Implications

In view of the above evidence, policy implication that will increase the probabilities of larger self-motivated adoption of CA technologies in the Jianghan Plain and beyond are proposed in the following aspects: (1) When landholders believe that innovation is beneficial to their personal goals, they will adopt it. According to our research results, we could promote farmers’ adoption of CA by enhancing their awareness of economic and social welfare. (2) Stronger measures should be implemented to cope with the flow and transfer of the farmland. As shown in the results, an appropriate scale of farmland essential for larger contracted area of farmland increased the possibility of adopting CA technologies. (3) Governments should put a greater focus on the development of rural factories, which will highly improve their ability to create and provide non-agricultural jobs for farmers. Only in this way would rural households have the foundation to adopt CA technologies in Jianghan Plain.

## Figures and Tables

**Table 1:** Variable definition and description.

Variable Types	Variable	Variable Definitions and Assignments
The dependent variable	CA adoption	Adopt five = 5; Adopt four = 4; Adopt three = 3; Adopt two = 2; Adopt one = 1; No adoption = 0
The independent variables	Full-time farmers	Non-agricultural income accounts for 0–10% = 1; others = 0
	I part-time farmers	Non-agricultural income accounts for 10–50% = 1; others = 0
	II part-time farmers	Non-agricultural income accounts for 50–90% = 1; others = 0
Individual characteristics	Gender	Male = 1; Female = 2
	Age	Actual value
	Education level	Primary school and below = 1; Primary school = 2; Junior high school = 3; High school = 4; University and above = 5
	To serve as a village cadre	Yes = 1; No = 2
Family characteristics	Number of agricultural labor force	Actual value
	Health condition of family members	Very poor = 1; Poor = 2; General = 3; Good = 4; Very good = 5
	Contracted area of farmland	Actual value
Welfare cognition	Economic welfare cognition	CA technology can bring me a good income: Totally disagree = 1; Disagree = 2; General = 3; Agree = 4; Totally agree = 5
	Social welfare cognition	CA technology is beneficial to social development and progress: Totally disagree = 1; Disagree = 2; General = 3; Agree = 4; Totally agree = 5
	Ecological welfare cognition	CA technology is good for ecological environment improvement: Totally disagree =1; Disagree = 2; General = 3; Agree = 4; Totally agree = 5
Region	Location of the respondents	Yicheng = 1, Jingshan = 2

**Table 2:** Basic social and economic conditions of the interviewed farmers.

Variable	Classification	Frequency	Proportion	Variable	Classification	Frequency	Proportion
Gender	Male	311	71.8%	Village cadres	Yes	38	8.8%
	Female	122	28.2%		No	395	91.2%
Age	≤40	20	4.6%	Annual family income (Yuan)	≤20,000 yuan	129	29.8%
	41–50	88	20.3%		>20,000–40,000	116	26.8%
	51–60	177	40.9%		>40,000–60,000	83	19.2%
	61–70	117	27.0%		>60,000–80,000	41	9.4%
	>70	31	7.2%		>80,000	64	14.8%
Education level	Primary school and below	63	14.5%	Contracted land scale	≤3 mu	43	9.9%
	Primary school	160	37.0%		>3–6 mu	76	17.6%
	Junior high school	181	41.8%		>6–9 mu	55	12.7%
	High school	26	6.0%		>9–12 mu	79	18.2%
	University and above	3	0.7%		>12 mu	180	41.6%

Note: 1 mu = 0.067 hm^2^.

**Table 3:** Socio and economic situation of different types of part-time households.

Part-time	Number	Proportion	Proportion Over	Junior High School	Annual Family Income	Contracted
Farming Situation		(%)	50 Years Old (%)	and Above (%)	(10 Thousand Yuan)	Land Area (mu)
Pure farmers	205	47.34	80.98	45.85	3.67	9.84
I part-time households	73	16.86	76.71	50.68	4.68	10.29
II part-time households	95	21.94	85.26	51.58	6.07	9.05
Non-agricultural households	60	13.86	81.67	50.00	8.53	7.50

Note: 1 mu = 0.067 hm^2^.

**Table 4:** Adoption rate of conservation agriculture (CA) technology.

Adoption	Number	Proportion	Proportion Over	Junior High School	Annual Family Income	Contracted
		(%)	50 Years Old (%)	and Above (%)	(10 Thousand Yuan)	Land Area (mu)
Yes	398	91.92	81.91	48.49	5.06	9.43

Note: 1 mu = 0.067 hm^2^.

**Table 5:** Results of the impact of part-time farming on the CA technology adoption.

Variable	Model I			Model II		
	Coefficient	SD	Exp(β)	Coefficient	SD	Exp(β)
Full time households (X_1_)	1.083 ***	0.304	2.953	0.698 *	0.365	2.011
I part-time households (X_2_)	0.959 ***	0.354	2.609	0.670 *	0.388	1.955
II part-time households (X_3_)	0.665 **	0.338	1.944	0.824 **	0.351	2.279
Gender (X_4_)				−0.822 ***	0.229	0.439
Age (X_5_)				−0.111	0.012	0.989
Education level (X_6_)				−0.004	0.121	0.996
Village cadre (X_7_)				−0.437	0.347	0.646
Number of agri-labor force (X_8_)				0.003	0.020	1.003
Health status (X_9_)				0.059	0.162	1.060
Contracted area (X_10_)				0.381 ***	0.130	1.463
Economic welfare cognition (X_12_)				0.005 *	0.026	1.048
Social welfare cognition (X_12_)				0.225 **	0.108	1.252
Ecological welfare cognition (X_13_)				0.169	0.142	1.184
Region (X_14_)				−0.730 ***	0.266	0.482
R^2^	187.676			500.294		
*p*	0.000			0.000		
Observations	433			433		

Note: ***, ** and * represent significant at statistical level of 1%, 5% and 10% respectively.
